# Physico-Chemical and Phytochemical Characterization of Moroccan Wild Jujube “*Zizyphus lotus (L.)*” Fruit Crude Extract and Fractions

**DOI:** 10.3390/molecules25225237

**Published:** 2020-11-10

**Authors:** Hafssa El Cadi, Hajar EL Bouzidi, Ginane Selama, Asmae El Cadi, Btissam Ramdan, Yassine Oulad El Majdoub, Filippo Alibrando, Paola Dugo, Luigi Mondello, Asmae Fakih Lanjri, Jamal Brigui, Francesco Cacciola

**Affiliations:** 1 Laboratory of Valorization of Resources and Chemical Engineering, Department of Chemistry, Abdelmalek Essaadi University, 90000 Tangier, Morocco; hafssa.elcadi@yahoo.fr (H.E.C.); hajarelbouzidi1995@gmail.com (H.E.B.); flasmaa@yahoo.fr (A.F.L.); jamalbrigui@yahoo.fr (J.B.); 2 Laboratory of Biochemistry and Molecular Genetics, Abdelmalek Essaadi University, 90000 Tangier, Morocco; ginane.selama@gmail.com; 3 Department of Chemistry, Laboratory of Physico-Chemistry of Materials, Natural Substances and Environment, Abdelmalek Essaadi University, 90000 Tangier, Morocco; asmae_cadi@yahoo.fr; 4 Laboratory of Biotechnology and valorization of natural resources, Department of Biology, Faculty of Science, University Ibn Zohr, 80000 Agadir, Morocco; ramdanbtissam8@gmail.com; 5 Department of Chemical, Biological, Pharmaceutical and Environmental Sciences, University of Messina, 98168 Messina, Italy; youladelmajdoub@unime.it (Y.O.E.M.); pdugo@unime.it (P.D.); lmondello@unime.it (L.M.); 6 Chromaleont s.r.l., c/o Department of Chemical, Biological, Pharmaceutical and Environmental Sciences, University of Messina, 98168 Messina, Italy; filippo.alibrando@chromaleont.it; 7 Department of Sciences and Technologies for Human and Environment, University Campus Bio-Medico of Rome, 00128 Rome, Italy; 8 BeSep s.r.l., c/o Department of Chemical, Biological, Pharmaceutical and Environmental Sciences, University of Messina, 98168 Messina, Italy; 9 Department of Biomedical, Dental, Morphological and Functional Imaging Sciences, University of Messina, 98125 Messina, Italy

**Keywords:** Rhamnaceae, phenolic compounds, flavonoids, tannins, antioxidant activity, liquid chromatography

## Abstract

Wild jujube “*Ziziphus lotus (L.) Desf*.” belongs to the Rhamnaceae family and is a traditionally herbaceous medicinal plant. It is very common in arid and semi-arid regions and is currently used for its antidiabetic, sedative, analgesic, anti-inflammatory and hypoglycemic activities. The aim of the present work was to characterize the physico-chemical properties and the phytochemical profile of wild jujube sample collected from the Guercif region, in order to determine the polyphenolic compounds and the antioxidant ability Analyses were carried out directly after the harvest for the determination of pH, refractive index, total soluble solid (°Brix), dry matter, sugar/acidity, total sugars, reducing sugars, as well as lipid and protein content. Results showed that the investigated fruit is acidic (pH 4.9 ± 0.23) and rich in sugars (80.2 g/100 g ± 3.81). The GC-MS analysis of the fruit revealed a number of volatile compounds, as many as 97, belonging to different chemical classes. The HPLC-DAD-ESI/MS analysis showed the presence of a total of 20 polyphenolic compounds in both EtOAc and MeOH-water extracts. Among them, *p*-Hydroxybenzoic acid was the most abundant in the EtOAc extract (185.68 µg/100 mg ± 0.5) whereas Quercetin 3-*O*-rhamnoside-7-*O*-glucoside was found in higher amounts in the MeOH-water extract (25.40 µg/100 mg ± 0.5). These components have medical interest, notably for human nutrition, as well as health benefits and therapeutic effects. Therefore, Moroccan jujube “*Zizyphus lotus (L.)*” fruit may have potential industrial applications for food formulations.

## 1. Introduction

Moroccan wild jujube (*Ziziphus lotus (L.) Desf*), widely called “Sedra” or “Nbag”, is found in several arid and semi-arid regions such as Chaouia, Haouz, Zear, Rhamna, the Middle Atlas Gharb, Errachidia, Souss, the coastal region of Safi in Sidi Ifni, Khenifra, eastern Morocco Sahara, and in the region of Oujda [1].

Jujube fruits are spherical drupes with a size of a pupil, and are eaten at full maturity in October. Their taste evokes candied apple and their texture is similar to dates. They are marketed for human consumption as a fermented drink by mixing crushed fruits with water, and as flour after drying it [2].

This species is known worldwide for many different medical uses e.g. its antipyretic and antiviral properties [3,4]. In antiquity, *Z. lotus* was used for its emollient properties; the mixture of dried leaves and fruits was applied topically in the treatment of boils and throat and bronchopulmonary irritations [5]. In previous studies, it has been also reported that *Z. lotus* root bark has anti-inflammatory, analgesic, and antidiabetic activities [6,7,8].

These fruits are famous for their high biologically active material contents such as polyphenols, exhibiting antioxidant, antimicrobial, and immunomodulatory properties [9]. Some studies carried out on butanol extracts of *Zizyphus* spina-christi leaves showed that they are rich in saponins and improve the glucose-induced insulin release in type II diabetic rats [10]. Moreover, *Z. lotus* aqueous and organic extracts are characterized by the presence of flavonoids and tannins [11]. Particularly, cyclopeptide alkaloids, termed lotusines, and dammarane saponins have been isolated from this shrub, along with polyunsaturated fatty acids (oleic acid and linoleic acid), carbohydrates, and fibers [12,13,14,15,16,17,18,19].

A comparative study of two *Zizyphus* species, namely *spina christi* and *lotus* from Morocco, highlighted the presence of essential nutrients and phytochemical compounds in the fruits, pulps, seeds, and almonds. Flavonoid and anthocyanin contents were found to be highest in methanolic extract of the seeds and almonds of *Z. lotus* [16]. An earlier study reported the concentrations of different vitamins (vitamin A, C, and E) and fatty acids in root, stem, leaves, fruit pulp, and seed of *Z. lotus* L. The results achieved showed higher vitamin A and C contents in the fruit pulp and a richer source of linoleic acid (18:2n−6) than that in other parts of the plant [20].

To the best of our knowledge, there is a lack of information about the physico-chemical properties, as well as the phytochemical composition of such a shrub growing wild in Morocco.; therefore, the present article will serve as an addition to the data that exists about Moroccan wild jujube. This work aims to evaluate the physico-chemical and bioactive properties of *Z. lotus (L.) Desf* fruit in order to potentially exploit them for industrial applications and incorporate them into food formulations to improve human health.

## 2. Results and Discussion

Nutritional quality of fruits is usually first characterized by physicochemical parameters, which can indicate a general estimate of the overall composition of their nutrient content. The most discriminating criteria are related to their sweet and sour taste but also their firmness. These are important factors in the sensory quality determination and the food products acceptability by consumers. In addition, the organoleptic quality is mainly determined by these chemical indicators. The phytochemical screening is required to detect the majority of compounds possessing essential biological activity.

### 2.1. Variation of Physicochemical Parameters

Jujube fruits are almost unknown to the majority of the Moroccan population, and there is lack of knowledge about their nutritional quality. Therefore, their physicochemical parameters have not been previously reported. The results achieved for refractive index, acidity, total soluble solid (°Brix), sugar/acidity, dry matter, ash, total sugars, and reducing sugars are shown in Table 1.

Considering the moisture content of the harvested fruits, a value of 12.9% was attained. This value does not fall within the range (58.34–76.5%) reported for other species of jujube (*Zizyphus jujuba* Mill.) present in China [21]. The moisture content is considered as a critical parameter to evaluate the quality of jujube fruits and can be considerably affected by genotype and culture conditions [22]. In general, the water content can be influenced by the age of the plant, the period of the vegetative cycle, and even genetic factors [20]. This variation may also be due to the different soil and climatic conditions and to the geographic distribution [23]. Other parameters studied by Chen et al. revealed the following values: ash content (0.8–1.1), total sugars (TS) (27.19–31.7), acidity (1.98–3.12), and sugar/acidity (S/A) (8.8–14.75) [21]. Studies on the same genus have found the percentage of dry matter values between these intervals: 7.88–77.93, 18.99–74.08, and 2.26–3.01, respectively, for % reducing sugars (RS), water content, and ash content. The same collected fruits in India revealed the following values, namely 1.4–6.2, 81.6–83, and 5.83, respectively, for RS, water content, and ash content [24]. The jujube fruits have a high total soluble solid (TSS) value, which is due to their high sugar content. This agrees with the results found by Zia-Ul-Haq et al. [25]. However, our values were different from those found by Gao et al. [26], probably due to the different extraction conditions. High quantities of TS were recovered from freeze-dried berries of jujube (80.2% ± 3.81). Cultivars of the Chinese jujube (*Zizyphus jujuba* cv) have shown values varying between 69.2% and 85.3% [27]. The average value of TS in the fractions was 6.23% ± 0.75 and 76.5% ± 1.21, respectively in EtOAc and MeOH-H_2_O fractions. The difference noted can be explained by the different polarity (*p* < 0.05) and the number of extraction cycles. There was a large gap between the values found in this study and those reported in China and India. This indicates that Moroccan jujubes, particularly from the Guercif region, have the sweetest character among the other studied jujubes. The soluble sugars of the Chinese jujube in five cultivars were identified as fructose, glucose, rhamnose, sorbitol, and sucrose [27]. Fructose and glucose were identified as the main sugars while sorbitol was present in small amounts. Other studies have also shown that glucose and fructose are the main present sugars [28]. The content of sucrose was found to be lower than the content of fructose and glucose. In fact, sucrose is synthesized in the leaves and is hydrolyzed to glucose and fructose with the invertase enzyme once translocated to the flesh of the fruit, which is known to occur during the ripening of the fruits [27]. TS content variations can be attributed to several factors such as the age of the plant, the burn load, the stage of ripening, and the fruit physiological state during analysis. Other factors such as the length of time in the sun, the climate, and the availability of water can also affect sugar content [21,29,30,31,32]. Indeed, a high concentration of sugars prevents bacterial proliferation in jams and jellies, and this contributes to the transformation of the studied fruit into several food products, in particular, jams, compotes, marmalades, and juice [24]. The average refractive index (RI) values were 2.8 ± 0.00 (EtOAc) and 2.7 ± 0.02 (MeOH-H_2_O). RI is influenced by the polarity of the solvents employed, as demonstrated by the ANOVA test (*p* < 0.05) [33]. Regarding protein content, a very low content was determined: 0.9 mg/g. The total concentrations of vitamin C were 34.5 mg/g ± 0.30, 12.7 mg/g ± 0.51, and 33.6 mg/g ± 0.45, respectively, for fresh fruits, EtOAc fraction, and MeOH-H_2_O fraction. The ANOVA test demonstrated a significant effect (*p* < 0.05) of solvent polarity on vitamin C content of jujubes.

### 2.2. Phytochemical Screening

The phytochemical screening of the wild jujube investigated in this work revealed the presence of different families of molecules, and results are presented in Table 2.

Results showed the presence of flavonoids, tannins, anthocyanins, coumarins, saponosides, sterols, deoxysugars, and mucilages. These results are in agreement with previous studies carried on the same species [34,35] and in a similar species, *Ziziphus mauritiana* [36]. Concerning polyphenols, the best solvent extraction was MeOH-H_2_O. For anthocyanins, only traces were detected in the MeOH-H_2_O fraction whereas they were totally absent in the EtOAc one. These results are in agreement with previous findings reported by Tiwari et al. [37]. Total tannins were the most abundant in the MeOH-H_2_O, as demonstrated by other studies [38,39,40]. Sterols and steroids were present in higher amounts in the MeOH-H_2_O fraction, in agreement with data obtained by Tiwari et al. [37]. Unsaturated sterols and terpenes were present in the EtOAc extract [39]. Saponosides were detected in the crude and MeOH-H_2_O extracts [37,39]; the same results were attained for mucilages [41].

Such a phytochemical prospecting of the studied fruits could be a good starting point for determining the presence of various classes of secondary metabolites [42,43,44,45,46,47].

Table 3 reports the quantification of total polyphenols (TPP), total flavonoids (TFv), total anthocyanins (TA), and total tannins (TT) content in *Z. lotus* solvent fractions. TPP was expressed as mg/g gallic acid equivalents (GAE), whereas flavonoid content was expressed in terms of mg/g quercetin equivalents (QE).

**Polyphenols:** For each fraction, the average value of polyphenols was 3.0 ± 0.10 mg GAE/g dry matter (DM) and 4.8 ± 1.05 mg GAE/g DM, respectively, for EtOAc and MeOH. The statistical analysis of variance test revealed that there was a significant difference between the levels of polyphenols in the fractions according to the extraction solvent used (*p* < 0.05) [48]. In many published reports, the most suitable solvent for polyphenols extraction was represented by ethyl acetate [49,50]; in the present work, the MeOH-H_2_O mixture yielded the highest content of polyphenols, in agreement with other works [51,52]. As a consequence, the recovery of polyphenols from plant materials was indeed influenced by the solubility of the polyphenolic compounds in the extraction solvent.

**Flavonoids:** The average value of TFv was 2.0 ± 0.10 mg QE/g DM and 5.7 ± 0.05 mg QE/g DM, respectively, for jujube fractions of EtOAc and MeOH-H_2_O. The difference between the two results was significant (*p* < 0.05). A study conducted on the phytochemical composition of sea buckthorn exposed a level of flavonoids ranging from 2.18 to 6.6 mg QE/g DM [53]. A similar result was found by Vinatoru et al. [54] who extracted flavonoids from carrot powder using ultrasound extraction. In another work carried out in Brazil, a research team found that acetonitrile could recover an optimal amount of Macela’s flavonoids “*Achyrolcine satureioides*” [55].

**Anthocyanins:** The mean value of TA for each fraction was equal to 0.1 ± 0.00 for both EtOAc and MeOH-H_2_O fractions. The statistical analysis of variance test (ANOVA) showed that there was a significant difference depending on the diversity between all the anthocyanin contents (*p* < 0.05). In Mexico, pure methanol showed the greatest capacity to extract TA from the skin of *Renealmia alpinia* fruit compared to acetonitrile [56]. Similar results were reported by Ju and Howard [57] where MeOH 60% showed a greater capacity than ethanol 60% and water for the extraction of TA and phenolic compounds from grape skin. In addition, Khonkarn et al. [58] pointed out that MeOH was the solvent with the highest yield of anthocyanins from coconut, rambutan, and mangosteen barks. The difference between the levels of anthocyanins in each solvent can be explained by the stability of the anthocyanins, which can react with the solvent present in the mother solution, as reported by Benabdeljalil et al. [59]. It is also important to point out that, on a theoretical level, anthocyanins increase with the ripening of the fruits [60].

**Tannins:** The average values obtained for tannin concentrations of EtOAc and MeOH-H_2_O fractions were, respectively, equal to 5.2 ± 0.01 µg catechin equivalents (CE)/g DM and 11.1 ± 0.50 µg EC/g DM. The MeOH-H_2_O solvent mixture turned out to be the best solvent for extracting the maximum level of tannins. Mokhtarpour et al. reported that using 50% aqueous MeOH, revealed high tannin levels compared to that of other treatments [61]. Ghasemi et al. [62] evaluated pistachio shells and attained maximum tannin content in the MeOH fraction. In addition, in a Moroccan study on *Acacia mollissima*, the best yield of tannins was observed for the MeOH-H_2_O fraction [63].

### 2.3. Antioxidant Activity

The mean IC_50_ of each solvent fraction studied showed that *Z. lotus* has the highest antioxidant power in the MeOH-H_2_O fraction (smallest IC_50_). Analysis by the ANOVA test showed a very significant difference between the results of the two fractions (*p* < 0.01). These values were lower than the ones by Najjaa et al. [64] but were higher than the ones reported by Ghazghazi et al. for methanol extracts [65]. 

Methanol has a great reduction capability and powerful free radical scavenging activity [66]. In this regard, in some medicinal plants, it has been found that the DPPH radical scavenging effects of methanolic extracts are greater than that of aqueous extracts. The same authors reported that the exhibited antioxidant activity by methanol extracts is due to the presence of phenolic substances such as rosmarinic acid from *Salvia officinalis* and *Origanum vulgare* [67]. Numerous in vitro studies have confirmed the ability of *Z. lotus* to scavenge free radicals and prevent cell damage [65,68]. In addition, it has been shown that *Z. lotus* do present antioxidant compounds belonging to different classes such as phenolic acids, flavonoids, and saponins. These components prevent oxidative stress by reducing reactive oxygen species (ROS), and a regular intake of natural antioxidants can lower the risk of various diseases by reducing oxidative stress [64].

### 2.4. GC-MS Analyses

**Figure 1:**
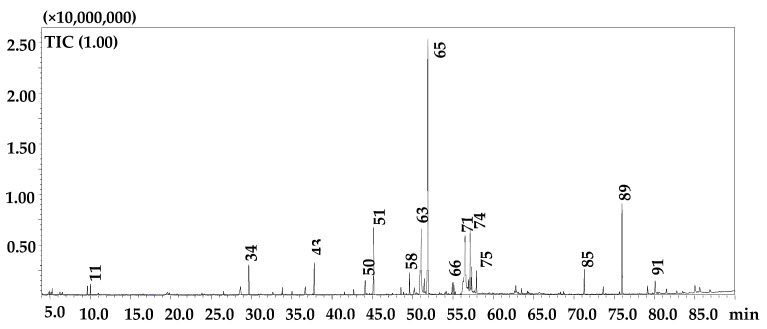
GC-MS profile of the *n*-hexane fraction of *Z. lotus.* Main peaks are labeled. Peak assignment as in Table 4.

The attained results of the GC-MS analysis of the *n*-hexane fraction showed the presence of lipids, alkanes, alcohols, sterols, and terpenoids. The studied fruits revealed a high number (N = 97) of these compounds as reported in Figure 1 and Table 4 with a % of similarity ranging from 83 to 97%.

### 2.5. HPLC-DAD-ESI/MS Analyses

**Figure 2:**
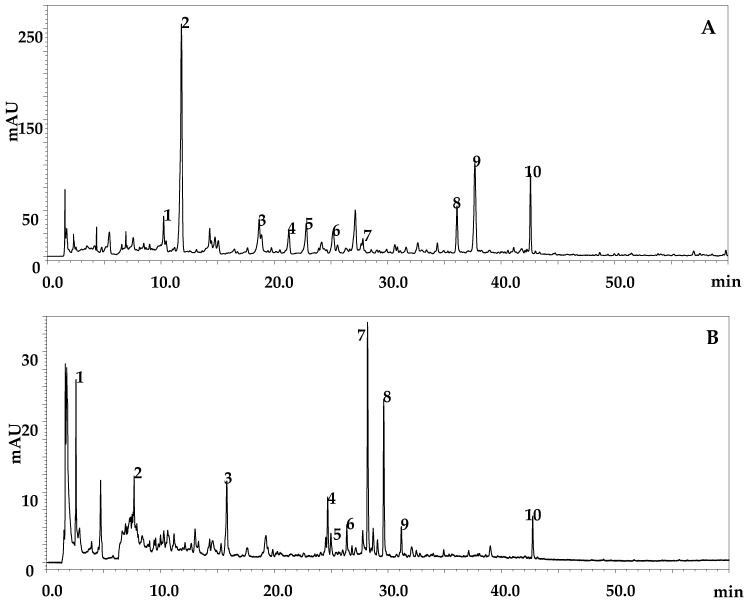
Polyphenolic profiles of *Z. lotus* extracts obtained by HPLC-PDA-ESI/MS analysis at λ = 280 nm: (**A**) EtOAc, (**B**) MeOH-H_2_O.

Figure 2 and Table 5 and Table 6 report the polyphenolic compounds identified in *Z. lotus* fruit extracts. A total of 20 different polyphenolic compounds were detected according to DAD, MS, and literature data. For the EtOAc extract, the phenolic compounds belonged to phenolic acid and derivatives and flavonols; in the MeOH-H_2_O extract, in addition to those compounds found in the EtOAc extract, organic acids and flavan-3-ols were detected. Considering phenolic acid and derivatives, the highest value was found for the EtOAc extract viz. 199.43 ± 0.8 vs. 2.42 ± 0.02 for the MeOH-H_2_O extract. Hydroxycinnamic acids were detected only in the EtOAc extract (84.69 ± 0.5); flavonols were more abundant in the MeOH-H_2_O extracts (31.99 ± 0.05 vs. 14.45 ± 0.01) (Table 7). These results are consistent with those of other studies carried out on the same species [20,35]. The difference in compounds detection can be due to their solubility in the extraction solvent, the degree of polymerization of the phenols, and the interaction of the phenols with other constituents of the plant [51,69].

## 3. Materials and Methods

### 3.1. Samples

Wild jujube fruits (*Z. lotus*) were collected for 4 months (May-June-July and August), all the harvest areas were between the longitude 3°38′13007 and the latitude 34°23′45746 of the Guercif region. The fruits were harvested at their physiological maturity in the early morning, transported in well-closed boxes, and stored at −10 °C in the Materials and Resources Valorization Laboratory, Faculty of Sciences and Technology of Tangier.

### 3.2. Chemical Reagents and Solvents

2,20-Diphenyl-1-picrylhydrazyl (DPPH), 2,20-azobis (2-amidinopropane), gallic acid dihydrochloride (AAPH), L-ascorbic acid, trichloroacetic acid (TCA), 1,1,3,3-tetraethoxypropane (TEP), thiobarbituric acid (TBA), and butylated hydroxytoluene (BHT) were purchased from Sigma (St. Lois, MO, USA). Folin-Ciocalteu phenol reagent was obtained from Fluka. Standards (gallic acid, cinnamic acid, rutin) were obtained from Merck Life Science (Merck KGaA, Darmstadt, Germany). LC-MS grade methanol, acetonitrile, acetic acid, acetone, and water were purchased from Merck Life Science (Merck KGaA, Darmstadt, Germany). All other chemicals were of analytical grade and obtained from Sigma (St. Louis, MO, USA).

### 3.3. Extraction Method

Five grams of lyophilized powder was defatted three times in 50 mL of *n*-hexane, dried, and homogenized with 50 mL of two solvents with increased polarity viz. EtOAc/water or MeOH/water (80:20 *v/v*) [70]. Each fraction was extracted by sonication in an ultrasound bath (130 kHz) for 45 min. The temperature was controlled by using a thermometer and gel ice box. After centrifugation for 5 min, the supernatant was filtered through a paper filter, dried, reconstituted with MeOH/water 80:20, *v/v,* and then filtered through a 0.45 μm Acrodisc nylon membrane (Merck Life Science, Merck KGaA, Darmstadt, Germany) prior to LC-PDA-MS analysis.

### 3.4. Physico-Chemical Analyses

The AOAC international standard methods 16 were used to determine the physico-chemical characteristics: ashes and DM content. The pH measurement was performed using a digital pH meter. Titratable acidity (TAc) was measured by the titrimetric method. RI and %TSS were determined by a digital refractometer.

### 3.5. Phytochemical Screening

Phytochemical screening was performed according to the method of Trease, E. and Evans, W.C. (1987). The tests were based on visual observation of the color change or the formation of a precipitate after addition of specific reagents [71].

### 3.6. Determination of the Polyphenolic Content

#### 3.6.1. Quantification of TPP, TFv, and TT in *Z. lotus* extracts

TPP content was estimated using the Folin–Ciocalteu method [72] with a few modifications. Gallic acid was used as the standard (10/25/50/100/200 ppm). TPP content was recorded at 755 nm and was expressed as mg of GAE/g of DM. Flavonoids were quantified according to the method of Zhishen et al. [29], using AlCl_3_ 10%, NaOH 4%, and NaNO_2_ 5%. The absorbance was determined at 510 nm. A curve of catechin was also carried out. TFv content was expressed as mg QE/g DM. TT content was determined by the vanillin method of Julkunen–Tiitto and expressed as mg CE/g DW [30].

#### 3.6.2. Quantification of Total Anthocyanin Content in *Z. Lotus* Extracts

TA content was estimated based on the differential pH (pH = 1 and pH = 4.5) by the method of Giusti and Wrolstad [31] with some modifications. Measurement was conducted at 510–700 nm in the UV-Vis spectrophotometer. The absorbance was calculated by the following formula:

A = [(A_510_ − A_700_) to pH 1.0] − [(A_510_ − A_700_) at pH 4.5]

The total anthocyanin content was calculated by the molecular weight of pelargonidine-3-glucoside using the following equation:

$$\left[\mathrm{mg}\;\mathrm{Pg}-3-\mathrm{glu}/g\;\mathrm{MS}\right]\;=\frac{A\;\times \;M\;\times \;F\;\times \;V\;\times \;1000}{\varepsilon \times d\times Q}$$

With M: molar mass of the pelargonidine-3-glucoside [g/mol], F: dilution factor, V: Volume of the extract (l), d: width of the Bowl (cm) and Q: quantity of homogenized fruit (g).

### 3.7. Determination of Antioxidant Activity

Free radical scavenging method DPPH(α, α-diphenyl-β-picrylhydrazyl) was carried out following the method described by Braca et al. [32] with minor modifications. Briefly, the fruit extracts were prepared from 25 µL of a methanolic solution and each of the pure compounds were added to 2 mL of DPPH (6.25 × 10^−5^ M). After gentle mixing and incubation for 30 min at room temperature in the dark, allowing for reactions to take place, the absorbance values of the resulting solutions were measured at 517 nm using a blank containing the same concentration of DPPH radicals. Inhibitions of DPPH radical in percent (I%) were calculated as follow:

I% = [(Abs_blank_ − Abs_sample_)/Abs_blank_] × 100

where A_blank_ is the absorbance of the control reaction (containing all reagents except the test compound) and A_sample_ is the absorbance of the test compounds. The sample concentration provided 50% inhibition (half-maximal inhibitory concentration, IC_50_) was calculated by plotting inhibition percentages against concentrations of the sample.

### 3.8. GC-MS

GC-MS analyses of volatile compounds was performed on an SLB-5ms column (30 m in length × 0.25 mm in diameter × 0.25 μm in thickness of film, Merck Life Science, Merck KGaA, Darmstadt, Germany). GC-MS detection involved an electron ionization system that utilized high energy electrons (70 eV). Pure helium gas (99.9%) was used as the carrier gas with a flow rate of 1 mL/min, and an injection volume of 0.7 μL was employed (a split ratio of 5:1). The initial temperature was set at 50 °C and increased up to 350 °C with an increase rate of 3 °C/min and holding time of about 5 min. Relative quantity of the chemical compounds present in each sample was expressed as percentage based on peak area produced in the chromatogram.

### 3.9. HPLC-DAD/ESI-MS

LC analyses were performed on a Shimadzu liquid chromatography system (Kyoto, Japan), consisting of a CBM-20A controller, two LC-30AD dual-plunger parallel-flow pumps, a DGU-20A5R degasser, a CTO-20AC column oven, a SIL-30AC autosampler, an SPD-M30A photo diode array detector, and an LCMS-8050 triple quadrupole mass spectrometer, through an ESI source (Shimadzu, Kyoto, Japan).

Chromatographic separations were carried out on 150 × 4.6 mm; 2.7 µm Ascentis Express RP C18 columns (Merck Life Science, Merck KGaA, Darmstadt, Germany). The mobile phase was composed of two solvents: water/acetic acid (99.85/0.15 *v/v*, solvent A) and acetonitrile/acetic acid (99.85/0.15 *v/v*, solvent B). The flow rate was set at 1 mL/min under gradient elution: 0–5 min, 5% B, 5–15 min, 10% B, 15–30 min, 20% B, 30–60 min, 50% B, 60 min, 100% B. DAD detection was applied in the range of λ = 200–400 nm and monitored at λ = 280 nm (sampling frequency: 40.0 Hz, time constant: 0.08 s). MS conditions were as follows: scan range and the scan speed were set at m/z 100–800 and 2500 u sec^−1^, respectively, event time: 0.3 sec, nebulizing gas (N_2_) flow rate: 1.5 L min^−1^, drying gas (N_2_) flow rate: 15 L min^−1^, interface temperature: 350 °C, heat block temperature: 300 °C, DL (desolvation line) temperature: 300 °C, DL voltage: 1 V, interface voltage: −4.5 kV. Calibration curves (R^2^ ≥ 0.997) of eleven polyphenolic standards used for the quantification in sample extracts were obtained using concentration (mg/mL) and according to the area of peaks at wavelengths of 270 nm, 277 nm, and 355 nm.

Compound identification was carried out by using complementary information coming from DAD, ESI-MS, and literature data.

### 3.10. Statistical Analysis

All analyses were performed in triplicate and data were reported as mean values and standard deviation (SD). The differences among treatments were detected by analysis of variance ANOVA (*p* < 0.05).

## 4. Conclusions

The present study shows that *Z. lotus* (L.) fruits possess interesting bioactive compounds as highlighted from the phytochemical profile. Results showed that the investigated fruits are acidic, rich in sugars, with a large array of volatile compounds belonging to different chemical classes. In addition, a total of 20 polyphenolic compounds were detected in both EtOAc and MeOH-water extracts. Such results demonstrate that *Z. lotus* (L.) is a potential source of bioactive compounds and can be potentially used for industrial applications in food formulations.

## Figures and Tables

**Table 1:** Different physico-chemical parameters of *Z. Lotus* samples. The results are expressed as mean ± standard deviation.

Parameter	Crude Extract	Solvent Fractions	
		EtOAc	MeOH-H_2_O
**pH**	4.9 ± 0.23	-	-
**Acidity**	1.5 ± 0.06	-	-
**RI**	1.3 ± 0.02	2.8 ± 0.00	2.7 ± 0.02
**TSS**	6.5 ± 0.92	60.8 ± 0.20	16.7 ± 0.48
**S/A**	4.2 ± 0.40	-	-
**DM (%)**	87.1 ± 0.25	-	-
**Ash (%)**	3.2 ± 0.54	-	-
**TS (%)**	80.2 ± 3.81	6.2 ± 0.75	76.5 ± 1.21
**RS (%)**	9.6 ± 0.39	-	-
**Lipids (mg/g)**	2.3 ± 0.09	-	-
**Proteins (mg/g)**	0.9 ± 0.02	0.9 ± 0.00	0.00
**Vitamin C (mg/g)**	34.5 ± 0.30	12.7 ± 0.51	33.6 ± 0.45

RI: refractive index; TSS: total soluble solid (°Brix); DM: dry matter; S/A: sugar/acidity; TS: total sugars; RS: reducing sugars.

**Table 2:** Phytochemicals detected in *Z. lotus* extracts.

Compounds Group/Solvent of Extraction		Crude Extract	EtOAc	MeOH-H_2_O
Alkaloids		-	±	±
**Polyphenols**	Flavonoids	C++	B	A+
	Tannins	+	-	++
	Anthocyanins	+	-	±
	Catechic tannins	+	-	+
	Gallic tannins	+	-	+
	Coumarins	+	-	-
**Steroids**	Soponosides	+	-	+
	Unsaturated Sterols/Terpenes	-	+	-
	Sterols and Steroids	++	-	++
**Sugars**	Deoxysugars	+	-	-
	Glycosides	-	+	±
	Mucilages	+	-	+

A: Flavone; B: Isoflavone; C: Flavonones; ++: Abundant; +: Presence of metabolite; -: Absence of metabolite; ±: trace.

**Table 3:** Total polyphenols (TPP), total flavonoids (TFv), total anthocyanins (TA), and total tannins (TT) content in *Z. lotus* solvent fractions.

Extract	Vit. C	TPP	TFv	TA	TT	IC_50_
**EtOAc**	12.7 ± 1.01	3.0 ± 0.10	2.0 ± 0.10	0.1 ± 0.00	5.2 ± 0.10	1.5 ± 0.00
**MeOH-H_2_0**	33.6 ± 2.50	4.8 ± 1.05	5.7 ± 0.05	0.1 ± 0.00	11.1 ± 0.50	1.3 ± 0.00

**Table 4:** List of compounds identified in *Z. lotus* by GC-MS.

Peak	Compound	LRI (lib)	LRI (exp)	Similarity (%)	Library
1	Isobutyric acid	752	740	83	FFNSC 4.0
2	3-Hexanone	782	781	93	FFNSC 4.0
3	Butyl methyl ketone	786	787	98	FFNSC 4.0
4	3-Hexanol	795	798	91	FFNSC 4.0
5	2-Hexanol	802	801	92	FFNSC 4.0
6	Isovaleric acid	842	838	97	FFNSC 4.0
7	2-methylbutanoic acid	881	849	94	FFNSC 4.0
8	*n*-Hexanol	867	867	88	FFNSC 4.0
9	*n*-Pentanoic acid	911	876	96	FFNSC 4.0
10	*n*-Heptanal	906	903	90	FFNSC 4.0
11	(*E*)-Hept-2-enal	956	957	93	FFNSC 4.0
12	*n*-Hexanoic acid	997	980	96	FFNSC 4.0
13	2-pentyl Furan	991	992	86	FFNSC 4.0
14	*n*-Octanal	1006	1004	91	FFNSC 4.0
15	Limonene	1030	1030	93	FFNSC 4.0
16	Oct-3-en-2-one	1036	1039	90	FFNSC 4.0
17	(*E*)-Oct-2-enal	1058	1059	93	FFNSC 4.0
18	*n*-Nonanal	1107	1105	96	FFNSC 4.0
19	methyl-Octanoate	1125	1124	93	FFNSC 4.0
20	Benzenecarboxylic acid	1213	1171	97	FFNSC 4.0
21	*n*-Octanoic acid	1192	1176	96	FFNSC 4.0
22	ethyl-Octanoate	1202	1196	95	FFNSC 4.0
23	*n*-Decanal	1208	1207	91	FFNSC 4.0
24	methyl-Nonanoate	1224	1224	88	FFNSC 4.0
25	(Z)-Dec-2-enal	1250	1250	89	FFNSC 4.0
26	(E)-Dec-2-enal	1265	1264	97	FFNSC 4.0
27	*n*-Nonanoic acid	1289	1270	94	FFNSC 4.0
28	ethyl-Nonanoate	1297	1295	93	FFNSC 4.0
29	Carvacrol	1300	1302	92	FFNSC 4.0
30	*n*-Undecanal	1309	1309	91	FFNSC 4.0
31	(E,E)-2,4-Decadienal	1322	1321	89	FFNSC 4.0
32	methyl-Decanoate	1327	1324	96	FFNSC 4.0
33	*n*-Decanoic acid	1398	1372	97	FFNSC 4.0
34	ethyl-Decanoate	1399	1395	97	FFNSC 4.0
35	methyl-Undecanoate	1423	1424	95	FFNSC 4.0
36	*n*-Undecanoic acid	1473	1466	95	FFNSC 4.0
37	ethyl-Undecanoate	1498	1494	96	FFNSC 4.0
38	ethyl 9-oxononanoate	-	1505	-	W11N17
39	methyl-Dodecanoate	1527	1524	96	FFNSC 4.0
40	isobutyl-Decanoate	1545	1545	92	FFNSC 4.0
41	*n*-Dodecanoic acid	1581	1566	96	FFNSC 4.0
42	butyl-Decanoate	1585	1586	88	FFNSC 4.0
43	ethyl-Dodecanoate	1598	1594	97	FFNSC 4.0
44	*n*-Tetradecanal	1614	1614	91	FFNSC 4.0
45	*n*-Tridecanoic acid	1668	1663	93	FFNSC 4.0
46	Apiole	1683	1679	92	FFNSC 4.0
47	Ethyl tridecanoate	-	1694	-	W11N17
48	Tridecyl methyl ketone	1697	1698	92	FFNSC 4.0
49	methyl-Tetradecanoate	1727	1725	97	FFNSC 4.0
50	*n*-Tetradecanoic acid	1773	1765	90	FFNSC 4.0
51	ethyl-Tetradecanoate	1794	1794	98	FFNSC 4.0
52	Hexadecanal	-	1818	-	W11N17
53	methyl pentadecanoate	-	1825	-	W11N17
54	Neophytadiene	1836	1837	93	FFNSC 4.0
55	Phytone	1841	1842	94	FFNSC 4.0
56	Pentadecylic acid	1869	1862	96	FFNSC 4.0
57	ethyl-Pentadecanoate	1893	1893	94	FFNSC 4.0
58	methyl (Z)-9-hexadecenoate	-	1904	-	W11N17
59	methyl (Z)-11-hexadecenoate	-	1913	-	W11N17
60	methyl-Hexadecanoate	1925	1926	96	FFNSC 4.0
61	9-Hexadecenoic acid	-	1944	-	W11N17
62	(*Z*)-11-Hexadecenoic acid	-	1953	-	W11N17
63	*n*-Hexadecanoic acid	1977	1971	95	FFNSC 4.0
64	Ethyl 9-hexadecenoate	-	1982	-	W11N17
65	ethyl-Palmitate	1993	1996	97	FFNSC 4.0
66	propyl hexadecanoate	-	2090	-	W11N17
67	ethyl heptadecanoate	-	2094	-	W11N17
68	methyl-Oleate	2098	2100	93	FFNSC 4.0
69	methyl-Octadecanoate	2127	2127	93	FFNSC 4.0
70	Linoleic acid	2144	2139	95	FFNSC 4.0
71	Oleic acid	2147	2142	90	FFNSC 4.0
72	(*Z*)-Vaccenic acid	-	2150	-	W11N17
73	ethyl-Linoleate	2164	2161	93	FFNSC 4.0
74	ethyl-Oleate	2166	2168	87	FFNSC 4.0
75	ethyl-Stearate	2198	2194	96	FFNSC 4.0
76	(*Z*)-9-Octadecenamide	-	2363	-	W11N17
77	hexyl hexadecanoate	-	2380	-	W11N17
78	ethyl-Eicosanoate	2394	2395	90	FFNSC 4.0
79	*n*-Tetracosane	2400	2400	87	FFNSC 4.0
80	*n*-Pentacosane	2500	2500	90	FFNSC 4.0
81	benzyl hexadecanoate	-	2581	-	W11N17
82	ethyl-Docosanoate	2595	2595	87	FFNSC 4.0
83	*n*-Hexacosane	2600	2600	90	FFNSC 4.0
84	ethyl docosanoate	-	2581	-	W11N17
85	*n*-Heptacosane	2700	2700	95	FFNSC 4.0
86	ethyl-Tetracosanoate	2796	2796	88	FFNSC 4.0
87	*n*-Octacosane	2800	2800	94	FFNSC 4.0
88	Squalene	2810	2814	87	FFNSC 4.0
89	*n*-Nonacosane	2900	2902	92	FFNSC 4.0
90	*n*-Triacontane	3000	3000	85	FFNSC 4.0
91	Octacosanal	-	3045	-	W11N17
92	10-Nonacosanone	-	3088	-	W11N17
93	*n*-Hentriacontane	3100	3100	92	FFNSC 4.0
94	Octacosanol	-	3111	-	W11N17
95	Vitamin E	-	3131	-	W11N17
96	Triacontanal	-	3250	-	W11N17
97	γ-Sitosterol	-	3323	-	W11N17

**Table 5:** Polyphenolic compounds detected in *Z. lotus* (EtOAc extract) by HPLC-DAD-ESI/MS.

Peak	Tentative Identification	t_R_ (min)	Identification Type	λ_MAX_ (nm)	*m/z*	Fragments
	**Phenolic acid and derivatives**					
1	synapic acid	10.23	DAD/MS	309	223	193, 161
2	*p*-Hydroxybenzoic acid	11.80	DAD/MS	254	137	-
4	p-coumaric acid	21.27	DAD/MS	308	163	-
5	*p*-Coumaroyl glucose	22.81	DAD/MS	293	325	163
6	benzoic acid	25.17	DAD/MS	273	121	-
9	cinnamic acid derivative	37.68	DAD/MS	277	650	616, 147
	**Flavonol**					
7	Rutin	27.80	DAD/MS	255–353	609	-
	**Not identified**					
3	Unknown	18.65	-	266	281	265+
10	Unknown	42.58	-	294–381	698	-
8	Unknown	36.10	-	264	263	-

**Table 6:** Polyphenolic compounds detected in *Z. lotus* (MeOH-H_2_O extract) by HPLC-DAD-ESI/MS.

Peak	Tentative Identification	t_R_ (min)	Identification Type	λ_MAX_ (nm)	*m/z*	Fragments
**Organic acid**						
1	Malic acid derivative	2.51	DAD/MS	-	503	191,133
	Phenolic acid and derivatives					
3	Galloyl shikimic acid	15.3	DAD/MS	252–286	325	
	**Flavan-3-ols**					
2	(-)-Catechin 3-*O*-gallate	7.35	DAD/MS	258	441	-
	**Flavonols**					
4	Quercetin rhamnosyl-rhamnosyl-glucoside	24.98	DAD/MS	253–357	755	303+
5	Quercetin di-glucoside	25.25	DAD/MS	254–357	625	303+
7	Quercetin rhamnoside-glucoside	28.53	DAD/MS	286	609	303+
8	Eriodictyol derivative	29.80	DAD/MS	285	597	287
**Non-identified**						
6	Unknown	26.51	DAD/MS	351	613	-
9	Unknown	31.31	DAD/MS	255–352	141	-
10	Unknown	43.02	DAD/MS	277–373	698	-

**Table 7:** Semi-quantification of polyphenols detected in *Z. lotus* fruits in µg/100 mg (*w*/*w*).

Compound	EtOAc	MeOH-Water	Standard Used
**Phenolic acid and derivatives**			
*p*-Hydroxybenzoic acid	185.7 ± 0.50	-	Gallic acid
benzoic acid	13.7 ± 0.50	-	Gallic acid
galloyl shikimic acid	-	2.4 ± 0.02	Gallic acid
**Total of Hydroxybenzoic acids**	**199.4 ± 0.80**	**2.4 ± 0.02**	
sinapic acid	60.0 ± 0.10	-	Cinnamic acid
*p*-coumaric acid	3.7 ± 0.04	-	Cinnamic acid
*p*-coumaroyl glucose	6.5 ± 0.01	-	Cinnamic acid
cinnamic acid derivative	14.5 ± 0.50	-	Cinnamic acid
**Total of hydroxycinnamic acid**	**84.7 ± 0.50**	**-**	
**Flavonols**			
Rutin	14.4 ± 0.01	-	Rutin
Quercetin rhamnosyl-rhamnosyl-glucoside	-	4.1 ± 0.02	Rutin
Quercetin di-glucoside	-	2.5 ± 0.05	Rutin
Quercetin rhamnoside-glucoside	-	25.4 ± 0.03	Rutin
**Total flavonols**	**14.4 ± 0.01**	**32.0 ± 0.05**	
